# Vitamin D levels and status amongst asthmatic and non-asthmatic adolescents in Cyprus: a comparative cross-sectional study

**DOI:** 10.1186/s12889-015-1385-2

**Published:** 2015-01-31

**Authors:** Ourania Kolokotroni, Anna Papadopoulou, Nicos Middleton, Christiana Kouta, Vasilios Raftopoulos, Polyxeni Nicolaidou, Panayiotis K Yiallouros

**Affiliations:** Cyprus International Institute for Environmental & Public Health in association with Harvard School of Public Health, Cyprus University of Technology, Limassol, Cyprus; Department of Nursing, School of Health Sciences, Cyprus University of Technology, Limassol, Cyprus; St George University of London Medical Programme at the University of Nicosia Medical School, Nicosia, Cyprus; 3rd Department of Pediatrics, Attikon University Hospital, University of Athens Medical School, Athens, Greece

**Keywords:** Adolescents, Asthma, Asthma severity, Vitamin D levels, Vitamin D status

## Abstract

**Background:**

Emerging evidence suggests that vitamin D might be implicated in asthma pathophysiology. This study aims to compare Vitamin D mean serum levels and status between asthmatic and non-asthmatic adolescents and investigate the association of vitamin D with asthma severity.

**Methods:**

In a cohort of adolescents aged 16–17 years, those reporting wheezing in the past 12 months and Ever asthma on the ISAAC questionnaire were invited to participate and formed the Active Asthmatics group. Controls were selected amongst Never Wheezers/Never Asthmatics (NWNA). Differences in mean 25(OH)D serum levels and vitamin D status between AA and NWNA were examined in multivariate linear and logistic regression models respectively, adjusting for potential confounders. Within AA, differences in vitamin D levels were assessed across asthma severity indicators.

**Results:**

A total of 69 AA and 671 NWNA participated in the study. Unadjusted mean 25(OH)D serum levels were 22.90 (SD 6.41), and 21.15 (SD 5.59) ng/mL in NWNA and AA respectively (p = 0.03). In adjusted models, mean 25(OH)D levels remained significantly lower amongst AA compared to NWNA (adjusted beta coefficient −1.68, 95% CI −3.24, −0.13). Severe (<12 ng/mL), moderate (<25 ng/mL) or insufficient (<30 ng/mL) vitamin D status was more prevalent among AA who were 1.6 times (95% CI 1.01, 2.53) more likely to belong to a lower vitamin D category compared to NWNA. Within AA, there was a negative trend between vitamin D levels and the number of reported asthma severity indicators.

**Conclusions:**

Levels of vitamin D tend to be lower among asthmatic compared to non-asthmatic children and in those with severe asthma independent of important confounders.

## Background

In the last decades, vitamin D deficiency has reappeared as a major public health problem worldwide affecting people of all ages, even in regions with abundant sun exposure [1-3]. Over the same period, the prevalence of asthma and allergies has been also increasing [4]. This has been mainly attributed to changes in environmental and lifestyle factors such as the reduced exposure to infections, dietary changes and obesity [5]. There is now an increasing body of evidence suggesting that vitamin D may have multiple biological effects, beyond bone metabolism, including the pathogenesis of respiratory and allergic diseases [6,7].

Recently, a fast rising number of studies have looked into a possible association between asthma and inadequate levels of vitamin D but evidence is still conflicting [8-18]. Some cross-sectional studies have shown serum vitamin D levels to be lower in asthmatic compared to healthy children [9,10,12,13] but others did not observe significant differences [11,14,15]. Even within the small number of prospective studies on the matter, findings are conflicting. For example, Van Oeffelen et al. found higher vitamin D levels at the age of four to be associated with reduced risk of asthma at the age of eight whilst Tolpannen et al. showed that higher 25(OH)D_3_ levels at the age of 10 are associated with increased risk of incident asthma and wheezing in the following year [16,17]. Additionally, there also seems to be limited and inconsistent findings regarding the role of vitamin D in the development of other allergic conditions [19,20].

A number of these studies were limited by their small sample sizes, the wide age range of study participants and differences in the definition of asthma [9,11,13,14]. Furthermore, a large number of studies reported only univariate comparisons of mean vitamin D levels between asthmatics and non-asthmatics [8,11,14], some matched cases and control for gender and/or ethnicity [9,10,12] but only few studies considered important confounders such as adiposity, sun exposure and season of testing [13,16]. Higher adiposity levels, lower sun exposure, winter season, female gender and darker skin types have been shown to be associated with lower vitamin D levels in healthy individuals due to their effect on vitamin D production and/or bioavailability [21,22]. Some of these factors such as obesity have been also shown to be more prevalent in asthmatic children [23]. Differences in these factors between asthmatic and non-asthmatic children can therefore obscure any true differences in vitamin D levels amongst the two groups.

Despite the so far controversial evidence on the association of vitamin D with asthma in the general population, findings from a small number of studies amongst asthmatics have shown an inverse relationship between sufficient vitamin D levels and asthma severity indicators such as the use of asthma medication [24,25], asthma exacerbations and hospitalisation in asthmatic children [8]. This could be suggestive of an important role of vitamin D in asthma development and/or management which needs further assessment.

The aim of this descriptive comparative population-based study was to compare mean vitamin D levels as well as the prevalence of hypovitaminosis between asthmatic and non-asthmatic adolescents in Cyprus, a country with rising childhood asthma and obesity [26,27] and investigate whether potential differences can be explained by the confounding effect of variables that relate to vitamin D production or bioavailability. Furthermore this study also assessed vitamin D levels in relation to asthma severity indicators.

## Methods

### Study population

Participants were selected from a cohort of 5384 adolescents aged 16–17 years who had completed the International Study of Asthma and Allergies in Childhood (ISAAC) questionnaire as part of a large school-based study on risk factors for asthma. All subjects who reported having wheezing in the past 12 months and ever asthma were invited to participate in the study and formed the active asthmatics (AA) group. Controls were selected amongst those adolescents who did not report either wheezing or asthma ever (Never Wheezers Never Asthmatics - NWNA). In line with the scope of another study on the relation of adiposity with asthma, NWNA had been selected using a stratified random sampling approach in order to increase the probability of selection of children at the extremes of BMI change between childhood and adolescence.

### Assessments

Participants attended the paediatric department of their nearest local hospital in the three main districts of the island (Nicosia, Limassol and Larnaca) between November 2007 and May 2008 and underwent blood sampling, anthropometric measurements and questionnaire assessments of physical activity levels, dietary intake of vitamin D and sun exposure as described previously [22].

In brief, Serum levels of 25-hydroxy-vitamin D were assessed using the enzyme immune assay kit of Immunodiagnostics Systems Ltd, UK (intra- and inter-assay coefficients of variation < 12%). Anthropometric measurements were taken in the morning with the subjects dressed in light clothing and without shoes. Body fat percent was measured with the use of a portable Body Mass Composition Analyser (TBF-300 Body composition Analyzer, Tanita Corporation, USA). The International Physical Activity Questionnaire (IPAQ) was employed in order to evaluate levels of physical activity and the data collected were analysed as per IPAQ guidelines [28]. Dietary Food Intake was assessed with the use of a Food Frequency Recall questionnaire (FFQ) which recorded the frequency of consumption of food items from 21 food categories over the preceding three months. Dietary intake of vitamin D was calculated based on the questionnaire data using local food-composition tables adapted to the Greek cuisine [29]. Sun exposure was reported by participants as time spent in the sun during weekends and holidays in the winter and in the summer months of the past 3 years recorded as hours per day in increments of 1 to a maximum of 4 hours per day [30]. In addition, the children’s habitual use of sun protection (use of sun block creams, clothing, hats) when out in the sun in the summer was recorded as never/rarely, occasionally, most of the time, and always/almost always. Furthermore, trained research assistants classified the child’s skin type as dark, olive, olive/medium, medium/fair and fair. Parental education as a proxy for socio-economic status (the highest level of educational attainment by either of the two parents), Current smoking status and Family history of allergies (father, mother, sibling- ever had asthma, eczema or allergic rhinitis) were also recorded. Finally the following asthma severity indicators were reported, based on the corresponding questions of the ECHRS questionnaire: Have you ever visited the Emergency department because you had difficulty with your breathing? (Emergency room visit), Have you ever been admitted to hospital with breathing problems, even for one night? (Hospital Admission), Have you had an asthma attack in the last 12 months? (Asthma attacks in the last 12 months), Are you currently on any medication for your asthma such as inhalers, liquid suspensions or oral tablets? (Asthma medication use). The study was approved by the Cyprus National Bioethics Committee and written informed consent was obtained from the children and their parents or guardians.

### Statistical analysis

Linear regression models were used to investigate (a) the association of 25(OH)D levels with a number of possible determinants within each study group and (b) differences in mean serum levels of 25(OH)D between the study groups before and after adjusting for potential confounders. Initial power calculations showed that the sample size had >90% power to detect a two-tail statistically significant result at the 5% level for 0.5 SD and 0.35 SD difference respectively (i.e. moderate to small) in vitamin D levels between the two comparison groups. Furthermore, odds ratios of inadequate vitamin D status between Active Asthmatics (AA) and the Non asthmatic group (NWNA) were estimated in binary logistic regression models where hypovitaminosis status was categorised as (a) severe deficiency (serum 25-(OH)D <12 ng/mL), (b) deficiency (<20 ng/mL) and (c) moderate Deficiency (<25 ng/mL) (d) insufficiency (<30 ng/mL). After ensuring that the proportional odds assumption holds, ordinal logistic regression analysis was used to estimate the proportional odds ratios across ordered vitamin D status categories. This provides a summary estimate and avoids the need to use estimates from different models to describe the relationship at each level of hypovitaminosis. Multivariable linear and logistic models adjusted for gender, body fat percent, self-reported sun exposure in winter, skin type and season of blood sampling since these were identified as important predictors for vitamin D levels in the NWNA group. While typically the annual seasonal variation of vitamin D levels displays a sinusoidal pattern, during our study which only spanned from November until May, this corresponded to a U-shape pattern which was similar in both cases and controls and, hence seasonality was controlled for by using a quadratic function i.e. month + month^2^. In all analyses, we applied normalised sampling weights based on inverse probability of selection of NWNA from the original population. Statistical analysis was performed using the statistical software PASW 18.

## Results

A total of 69 AA and 671 NWNA participated in the study with 63% and 72% response rates respectively of the populations that were targeted for recruitment. Baseline characteristics of participants in each group are presented in Table 1. The mean age of participants (of whom 1 in 4 were male) was 17 (SD 0.6) years. The study groups did not differ significantly in most characteristics including age, gender, parental education level, physical activity levels, dietary intake of vitamin D, use of sun protection or skin type. Interestingly, controls reported lower levels of sun exposure both during the summer as well as winter and AA reported higher frequency of current smoking despite differences not reaching statistical significance. Specifically, only 17.4% of the NWNA reported exposure to the sun for more than 3 hours per day during holidays and weekends in the winter as opposed to 24.6% of the AA whilst the frequency of current smoking was 15.9% in AA as compared to 9.8% in NWNA (Table 1). As expected reporting of a positive family history of allergies was significantly higher in AA as compared to NWNA (49.3% vs 19.4%, p = 0.00). Finally a higher proportion of NWNA had their vitamin D levels measured during the winter season as compared to AA and CWO.

**Table 1:** **Participant characteristics by study group**

	***Never Wheezers Never Asthmatics (NWNA)***	***Active Asthmatics (AA)***	***p-value*** ^***†***^
	***n = 671***	***n = 69***	
*Gender:* Male	42.8%	43.5%	0.91
*Season of assessments*			
Autumn	11.3%	2.9%	0.00
Winter	50.1%	31.9%	
Spring	38.6%	65.2%	
*Parental education level*			
None/Primary	3.0%	7.2%	0.14
Secondary	61.9%	55.1%	
Tertiary	35.2%	37.7%	
*Sun exposure in the Summer*			
< 1 hour/day	2.8%	4.3%	0.22
1-2 hours/day	15.8%	7.2%	
2-3 hours/day	25.9%	23.2%	
3-4 hours/day	24.8%	24.6%	
>4 hours/day	30.6%	40.6%	
*Sun exposure in the Winter*			
< 1 hour/day	21.6%	15.9%	0.55
1-2 hours/day	39.2%	39.1%	
2-3 hours/day	21.9%	20.3%	
3-4 hours/day	11.4%	17.4%	
>4 hours/day	6.0%	7.2%	
*Sun protection*			
Never/Rarely	24.7%	24.6%	0.47
Occasionally	37.2%	39.1%	
Most of the times	21.5%	14.5%	
Always/Almost always	16.6%	21.7%	
*Skin type*			
Olive	4.0%	2.9%	0.97
Olive medium	69.5%	69.6%	
Medium fair	25.0%	26.1%	
Fair	1.5%	1.4%	
*Exercise level (IPAQ)*			
Low	52.9%	49.2%	0.40
Moderate	34.0%	41.5%	
High	13.1%	9.2%	
*Dietary intake vitamin D*			
<400 IU	93.9%	91.2%	0.37
*Family history of allergies*	19.4%	49.3%	<0.01
*Current smokers*	9.8%	15.9%	0.11
*Age* **–** Mean (SD) in years)	17.0 (0.61)	16.9 (0.57)	0.63
*Body fat percent* – Mean (SD)	22.0 (8.9)	21.7(8.6)	0.77

^***†***^Notes: p-value of t-test in the case of age and body fat percent; otherwise, p-value of chi-square test.

Levels of 25(OH)D appeared to be normally distributed in the study populations whilst mean values were generally low in both groups (Table 2). With a mean of 21.15 ng/ml (SD 5.59), levels were lower among AA as compared to 22.90 ng/ml (SD 6.41) in NWNA. Among the control group, female gender, season of blood testing, lower sun exposure in winter, darker skin type and higher body fat percent were significantly associated with lower vitamin D levels (Table 2). In fact, a similar pattern was observed within AA, which however did not always reach significance at the 5% level.

**Table 2:** **Differences in mean 25(OH)D by participant characteristics in the two study groups (NWNA and AA) separately**

		**Never Wheezers Never Asthmatics (NWNA)**		**Active Asthmatics (AA)**	
		**n = 671**		**n = 69**	
Variable		*Mean 25(OH)D levels (SD)*	*p-value* ^*†*^	*Mean 25(OH)D levels (SD)*	*p-value* ^*†*^
*Gender*	Male	23.87 (6.62)	<0.01	21.70 (5.20)	0.48
	Female	22.23 (6.19)		20.71 (5.92)	
*Season*	Autumn	25.86 (6.73)	0.03	26.80 (2.55)	0.49
	Winter	22.73 (6.51)		20.91 (5.43)	
	Spring	22.61 (6.10)		21.01 (5.72)	
*Sun exposure in Summer*	<1 hour	22.05 (6.05)	0.25	16.33 (6.23)	0.09
	1-2 hours	22.58 (7.13)		21.08 (8.45)	
	2-3 hours	22.57 (6.25)		19.78 (5.97)	
	3-4 hours	23.07 (5.88)		21.94 (4.47)	
	>4 hours	23.22 (6.63)		22.00 (5.34)	
*Sun exposure in Winter*	<1 hour	22.16 (6.62)	<0.01	17.63 (6.18)	0.18
	1-2 hours	22.31 (6.06)		22.34 (5.100	
	2-3 hours	23.69 (6.67)		20.01 (5.53)	
	3-4 hours	23.98 (5.83)		22.68 (6.12)	
	>4 hours	25.13 (7.52)		22.18 (3.20)	
*Use of sun protection*	Never/rarely	22.75 (6.10)	0.36	20.49 (6.99)	0.94
	Occasionally	22.88 (6.88)		22.11 (4.42)	
	Most of the time	22.16 (5.79)		18.69 (5.14)	
	Almost always/Always	23.93 (6.54)		21.73 (6.08)	
*Skin type*	Olive	23.42 (8.84)	0.04	23.95 (0.78)	0.01
	Olive/Medium	22.48 (6.23)		22.15 (5.00)	
	Medium/Fair	23.83 (6.59)		18.47 (6.55)	
	Fair	24.30 (5.37)		16.30 (0)	
*Exercise level (IPAQ)*	Low	22.75 (6.57)	0.40	21.13 (6.14)	0.81
	Moderate	22.88 (6.23)		21.06 (5.25)	
	High	23.47 (6.07)		22.05 (5.27)	
*Parental education*	Elementary	22.68 (7.44)	0.62	22.20 (3.05)	0.87
	Secondary	22.99 (6.50)		20.80 (5.38)	
	Tertiary	22.65 (6.16)		21.48 (6.28)	
*Smoking status*	No	22.86 (6.75)	0.06	21.27 (5.68)	0.69
	Yes	24.54 (6.83)		20.52 (5.36)	
*Body fat percent*	Quartile 1	23.48 (6.30)	0.02	22.23 (5.68)	0.33
	Quartile 2	23.42 (7.08)		21.45 (4.66)	
	Quartile 3	23.07 (5.91)		20.72 96.17)	
	Quartile 4	21.78 (6.23)		20.38 (6.08)	
*Dietary intake vitamin D*	<400 IU	22.96 (6.43)	0.13	20.87 (5.59)	0.14
	>400.1 IU	21.27 (5.77)		24.90 (6.83)	

Notes: ^†^With the exception of gender and dietary vitamin D intake where p-value for difference is reported, in all other cases, p-value for linear trend is reported.

Differences in mean 25(OH)D levels and Vitamin D status between the two groups are presented in Table 3. AA had significantly lower mean 25(OH)D levels when compared to NWNA (adjusted b coefficient −1.67, 95% CI −3.20, −0.14, p = 0.03). The observed differences were small (in the order of 2–3 ng/ml) but remained statistically significant even after adjusting for gender, season of testing, body fat percent, sun exposure and skin type in multivariable linear regression models.

**Table 3:** **Differences in mean vitamin D levels and prevalence of inadequate vitamin D status between asthmatic and non-asthmatic participants**

		***Mean (SD)***	***Unadjusted Model*** ^***†***^	Adjusted Model*
			***b coefficient (95% CI)***	***b coefficient (95% CI)***
*Vitamin D levels*	*NWNA*	22.90 (6.41)	Ref	Ref
	*AA*	21.15 (5.59)	−1.76 (−3.34 , −0.17); p = 0.03	−1.67 (−3.20, −0.14); p = 0.03
		*Prevalence (%)*	*Unadjusted Model* ^*‡*^	Adjusted Model*
			*OR (95% CI)*	*OR (95% CI)*
*Severe vitamin D deficiency*	*NWNA*	4.0%	Ref	Ref
*(levels < 12 ng/mL)*	*AA*	8.7%	2.32 (0.92, 5.84); p = 0.08	2.54 (0.97, 6.62); p = 0.06
*Vitamin D deficiency*	*NWNA*	34.7%	Ref	Ref
*(levels <20 ng/mL)*	*AA*	40.6%	1.26 (0.76, 2.10); p = 0.37	1.21 (0.72, 2.04); p = 0.47
*Moderate vitamin D deficiency*	*NWNA*	61.6%	Ref	Ref
*(levels <25 ng/mL)*	*AA*	75.4%	1.88 (1.05, 3.37); p = 0.03	1.91 (1.05, 3.49); p = 0.04
*Vitamin D insufficiency*	*NWNA*	84.0%	Ref	Ref
*(levels < 30 ng/mL)*	*AA*	94.2%	3.45 (1.06, 11.22); p = 0.04	3.23 (0.98, 10.65); p = 0.05
*Odds of belonging to a lower category of vitamin D levels* ^*#*^	*NWNA*		Ref	Ref
	*AA*		1.60 (1.01, 2.51); p = 0.04	1.60 (1.01, 2.53); p = 0.04
p value for proportionality assumption in the ordinal model			p = 0.509	p = 0.656

Notes: ^†^b coefficients corresponds to difference in mean vitamin D levels between Active Asthmatics (AA) and the Non asthmatic group (NWNA) as estimated in linear regression models before and after adjusting for potential confounders ^‡^ Odds ratios of inadequate vitamin D status between AA and NWNA as estimated in logistic regression models before and after adjusting for potential confounders *Multiple linear and logistic regression models adjusted for gender, body fat percent, season of assessment, self-reported sun exposure in winter, skin type. ^#^Estimated in ordinal logistic regression models along with p-value of test for parallel lines; a non-significant value provides evidence that the proportionality assumption of the ordinal model holds.

Overall, as many as 1 in 3 participants were vitamin D deficient (i.e. levels <20 ng/ml) whilst the prevalence of vitamin D insufficiency (i.e. <30 ng/ml) exceeded 80%. Generally higher prevalence of inadequate vitamin D status was observed among AA compared to non-asthmatics, although differences were statistically significant only with regards to moderate deficiency (<25 ng/ml) and insufficiency (<30 ng/ml ). AA were 3-times more likely to have 25(OH)D levels lower than 30 ng/ml (OR 3.45, 95% CI 1.06, 11.22) and 2-times more likely to have 25(OH)D levels lower than 25 ng/ml (OR 1.88, 95% CI 1.05, 3.37). After adjusting for factors that may confound the observed associations, there was generally not much attenuation in the estimates even though the odds ratio estimate remained statistically significant only for moderate vitamin D deficiency (OR 1.91, 95% CI 1.05, 3.49). Furthermore, using an ordinal logistic regression model to provide a summery estimate of the odds ratio across ordered categories of decreasing 25(OH)D levels (i.e. < 12, < 20, < 25, < 30 ng/ml), AA appeared to be 1.6- times (95% CI 1.01, 2.53) more likely to belong to a lower status of vitamin D compared to NWNA (p-value for test of the proportionality assumption in the ordinal logistic model = 0.67).

Finally, in Table 4, differences in vitamin D levels across asthma severity indicators in the AA group are presented. In general, participants reporting positively to any of the asthma severity questions had lower, albeit not always significant, levels of vitamin D. More specifically, children reporting having at least one asthma attack in the last 12 months and ever needing to use the emergency department for breathing difficulties had significantly lower vitamin D levels by 3–4 units as compared to those that responded negatively to these questions (b coefficient −3.05, 95% CI −6.07, −0.04 and b coefficient −3.76, 95% CI −6.74, −0.79). Similarly, lower levels of vitamin D, albeit of lower magnitude and not significant were noted in children reporting asthma medication use and ever needing a hospital admission for breathing problems (b coefficient −1.80, 95% CI - 4.82, 1.22 and b coefficient −2.40, 95% CI −6.13, 1.34). More importantly, a linear negative trend between vitamin D levels and the number of reported asthma severity indicators was noted. Specifically, for every one additional positive response to the asthma severity questions there was a significant decrease in vitamin D levels (b coefficient −1.55, 95% CI −2.78, −0.33).

**Table 4:** **Difference in Vitamin D levels by asthma severity indicators in active asthmatics**

***Asthma severity indicators***		***Vitamin D***	***Difference in mean Vitamin D levels between negative and positive response***	
		***Mean levels***		
		***Ng/mL (SD)***	***(Reference Group: Negative)*** ^***§***^	
		***n = 69±***	***b coefficient (95% CI); p value***	
			Model 1*	Model 2*
*Asthma attacks in the last 12 months*				
No	59.4%	21.58 (5.54)	−3.10	−3.05
Yes	40.6%	19.41 (5.40)	(−5.80, −0.41); p = 0.03	(−6.07, −0.04); p = 0.05
*Asthma medication use*				
No	62.3%	21.38 (5.31)	−2.00	−1.80
Yes	37.7%	19.35 (5.78)	(−4.92, 0.92); p = 0.18	(−4.82, 1.22); p = 0.24
*Hospital admission*				
No	82.2%	21.07 (5.28)	−2.90	−2.40
Yes	18.8%	18.52 (6.45)	(−6.18, 0.67); p = 0.11	(−6.13, 1.34); p = 0.20
*Emergency room visit*				
No	65.2%	21.77 (5.04)	−3.43	−3.76
Yes	34.8%	18.72 (5.89)	(−6.18, −0.67); p = 0.02	(−6.74, −0.79); p = 0.01
*Asthma severity index*				
No Positive Answers	29.5%	22.62 (4.84)	−1.51^†^	−1.55^†^
1 Positive Answer	26.2%	21.13 (5.57)	(−2.64, −0.37); p = 0.01	(−2.78, −0.33); p = 0.01
2 Positive Answers	23.0%	19.75 (5.28)		
3 Positive Answers	16.4%	18.50 (6.18)		
4 Positive Answers	4.9%	16.43 (6.58)		

Notes: ^§^b coefficients correspond to difference in mean vitamin D levels as estimated in linear regression models. *Model 1adjusted for seasonality. Model 2 further adjusting for gender, family history of Allergies, body fat %, smoking status and parental education. ±Missing data ranged from 0–8. ^†^Change in mean vitamin D levels per 1 unit change in asthma severity index (i.e. across increasing categories of severity).

## Discussion

In this study, there was evidence to suggest that active asthmatics have generally lower 25(OH)D levels and are more likely to belong to a lower vitamin D status category across the range of 25(OH)D values, which appears to be independent of factors that relate to the production or bioavailability of vitamin D. Within Active asthmatics, lower vitamin D levels were associated with asthma severity indicators such as asthma attack in the last 12 months and emergency room visit for breathing problems. Of note is the very high proportion of asthmatics with vitamin D deficiency and insufficiency in our study, even against a background of a generally high prevalence of vitamin D deficiency in Cyprus. This is however comparable with vitamin D status of asthmatic children in the region as also seen in the study by Chinelato et al. in Italy [11].

There are a number of limitations. The cross-sectional design of the study does not permit any causal inference with regards to the role of vitamin D in the development of asthma. Nevertheless, unlike a number of previous published studies, we were able to examine and adjust for the effects of a number of factors that could potentially confound the observed differences in vitamin D levels between asthmatics and non-asthmatics. Even though there was not much attenuation in the fully adjusted estimates, we cannot exclude the presence of residual confounding nor the fact that some of the confounding variables were measured with the use of self-reported questionnaires (e.g. sun exposure). Thus, while exposure misclassification is possible, there is no reason to suspect that this should be differential between the comparison groups.

Evidence from this study suggesting that active asthmatics have significantly lower 25(OH)D and a more compromised vitamin D status compared to non-asthmatic children comes to add to the somewhat conflicting evidence that has so far come from previous studies on this topic [8-15]. Nonetheless, our findings seem to be more consistent with those from previous studies that also considered potential confounders either at the design or analysis stage [9,10,12,13]. For example, two studies from the Middle East showed significant differences in mean vitamin D levels between asthmatic and non-asthmatic children matched by gender [9] and ethnicity [10,12]. Similarly in the study by Freishtat et al. from Washington DC, USA median vitamin D levels were significantly lower in children with physician diagnosis of asthma compared to non-asthmatic controls, whilst the prevalence of vitamin D deficiency and insufficiency was significantly higher in asthmatics even after adjusting for age, sex, BMI and season of sampling [13]. In contrast, the studies that didn’t find any evidence of differences in vitamin D levels between asthmatics and controls [11,15], or even showed that vitamin D levels were higher amongst asthmatics than healthy controls [8], tend to provide only univariate comparisons.

Indeed, differences in the distribution of predictors of vitamin D levels amongst asthmatic and non-asthmatic children might exaggerate or mask a true difference in vitamin D concentrations between the two groups. Obesity for example has been shown to be more prevalent between asthmatics [23,31] but also associated with lower levels of vitamin D due to its sequestration in body fat stores [32]. Similarly, asthma and vitamin D deficiency are both more prevalent in female adolescents as opposed to male [21,33]. Tolppanen et al. [16] In this study, however, we showed that the observed differences in vitamin D levels and status between asthmatic and non-asthmatic children appear to be independent of differences in confounders such as gender, body fat percent, sun exposure in winter, skin type and season of blood sampling between the groups. In fact, further adjusting for other potentially important predictors of vitamin D levels, such as diet or physical activity, does not alter this pattern.

Nevertheless, at about 2 ng/mL, the magnitude of the observed difference in average levels of 25(OH)D between asthmatics and non- asthmatics in our study appears to be small, and thus, one could question its clinical significance. On the other hand, we showed that differences in the vitamin D status, as defined by cut off levels used for bone metabolism are much larger in magnitude. Irrespective of the cut-off point used, active asthmatics appeared up to 1.6 times more likely to belong to a lower vitamin D category. Furthermore, active asthmatics appeared 2 to 3-times more likely to have 25(OH)D levels under 30 ng/ml (insufficiency) as well as 12 ng/ml (severe deficiency) compared to non-asthmatic adolescents, while this difference was less evident in terms of vitamin D deficiency (i.e. 20 ng/ml). In general, the study had 80% power to detect an association in the magnitude of OR > 2. Since low levels of vitamin D were generally observed even among children in the control group (i.e. large concentrations of participants around values of 20–25 ng/ml), a larger sample would have been required to detect a statistically significant difference between the observed 41% Vs 35% in the prevalence of vitamin D deficiency in AA and NWNA respectively. Nevertheless, the magnitude of the observed prevalence difference was much larger and thus statistically significant at other critical values of the vitamin D range.

Furthermore, the results of this study showed that reporting of asthma severity indicators and specifically “having an asthma attack in the last 12 months” or “attending the emergency department with breathing difficulties” were associated with significantly lower vitamin D levels. These findings are consistent with most previous published studies which have shown fewer asthma exacerbations and lower utilisation of health care facilities for urgent treatment in asthmatic children with higher vitamin D levels [8,9,24,34]. Lower vitamin D levels have also been shown to be associated with higher asthma medication use and/or hospital admission for asthma [24,25]. The association of vitamin D with these indicators, despite not reaching statistical significance in our study has also been shown to be in the same direction.

A number of biological explanations have been proposed for the role of vitamin D in asthma pathogenesis. Vitamin D, through its receptors on immune cells, has been shown to promote the activities of innate immunity whilst suppressing those of adaptive immunity, and thus protecting from microbial invasion whilst down regulating lung inflammation [35]. Furthermore vitamin D has been implicated in cell differentiation and airway remodelling through its receptors on epithelial cells lining the respiratory tract and bronchial smooth muscle cells [36]. These mechanisms could potentially explain how individuals with lower vitamin D levels might be at higher risk of developing asthma or having a more severe form of the disease [6]. On the other hand it is possible that asthma and vitamin D deficiency share the same genetic loci resulting in merely the co-existence of the two conditions in affected individuals as in the case of different variants of vitamin D receptors on immune cells or cells of the respiratory tract of asthmatics compared to non-asthmatic individuals [36,37].

## Conclusions

This study has showed that asthmatic children have lower mean vitamin D levels and an increased likelihood of belonging to an inadequate vitamin D status, which does not seem to be explained by differences in behavioural or other factors implicated either in vitamin D production or bioavailability. Furthermore, that lower vitamin D levels are associated with the presence of asthma severity indicators. There is still much need for more longitudinal studies to investigate the effects of vitamin D deficiency on asthma development and severity as well as a better understanding of the biological mechanisms behind this association. Furthermore, interventional studies looking at the potential beneficial effects of vitamin D supplementation will shed more light on the reversibility of the vitamin D- asthma relationship and consequently on the causal association of vitamin D with asthma.
